# Supplementary material for: Perceived deservingness shapes attitudes toward environmental migrants in rural Bangladesh
Source: Commun Earth Environ. 2026 Feb 26;7(1):247. doi: 10.1038/s43247-026-03320-6 (PMC12995714; doi:10.1038/s43247-026-03320-6)
Supplement: Supplementary file 2 — Supplementary Information PDF file [file 43247_2026_3320_MOESM2_ESM.pdf]

Supplementary Information for ‘Perceived deservingness shapes attitudes toward environmental migrants in rural Bangladesh’

Lukas Rudolph (University of Konstanz), Linus Hormuth (University of Zurich), Jan Freihardt (ETH Zurich), and Vally Koubi (ETH Zurich)

Communications Earth & Environment

January, 2026

A Supplementary Methods **2**

A.1 Survey information 2

A.2 Summary statistics 3

B Supplementary Discussion **4**

B.1 Descriptive migrant attitudes including prime treatments 4

B.2 Additional conjoint experimental estimates and supporting information . 7

C Supplementary Note **20**

C.1 Deviations between pre-registration and article 20

C.2 Deviations between actual pictorial conjoint implementation and article description thereof 20

A Supplementary Methods

A.1 Survey information

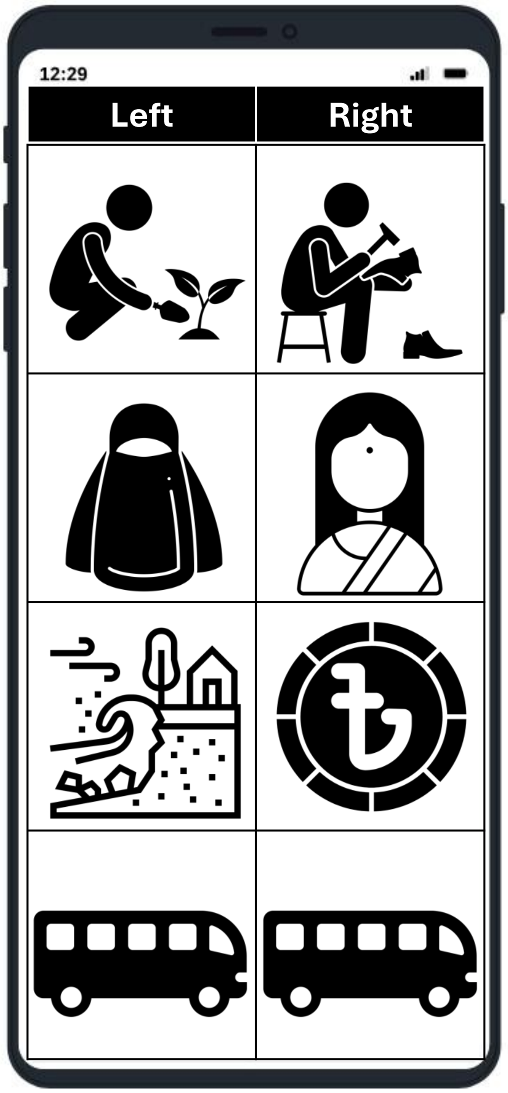

Supplementary Figure A.1: Example of a conjoint table as shown to survey respondents. Note that, for reasons of publication under a CC-BY license, the icons are visually distinct but conceptually equivalent to what was shown to respondents (see Supplementary Note C.2 for details). Icons are sourced from 'The Noun Project' (compare Figure 5) and licensed under CC-BY 3.0. Artist names are (from top left to bottom right): Top left: Gan Khoon Lay; top right: Gan Khoon Lay; upper middle left: Izwar Muis; upper middle right: Lufti Gani Al Achmad; lower middle left: WiStudio; lower middle right: BEARicons; lower left and lower right: Adrien Coquet. Links to the icons are available in Supplementary Note C.2.

A.2 Summary statistics

	Full Sample		Conjoint Sample	
Variable	Mean	SD	Mean	SD
Female	0.52	0.50	0.53	0.50
Age	44.62	14.20	43.03	14.72
Education				
No education	0.58	0.49	0.59	0.49
Primary education	0.20	0.40	0.23	0.42
Secondary education (or higher)	0.19	0.39	0.15	0.36
Muslim	0.96	0.19	0.97	0.16
Number of household members	4.90	1.98	4.97	1.83
Income				
No income	0.11	0.32	0.14	0.35
1 to 2000 taka	0.00	0.06	0.01	0.08
2001 to 6000 taka	0.17	0.37	0.21	0.41
6001 to 10000 taka	0.19	0.39	0.17	0.37
10001 to 14000 taka	0.12	0.33	0.09	0.29
14001 to 18000 taka	0.07	0.26	0.08	0.28
18001 to 22000 taka	0.08	0.27	0.09	0.29
22001 to 26000 taka	0.03	0.16	0.02	0.14
26001 to 30000 taka	0.01	0.11	0.01	0.08
more than 30000 taka	0.05	0.22	0.06	0.23
Household income enough to sustain livelihood	0.38	0.49	0.36	0.48
Here since birth	0.45	0.50	0.45	0.50
Lost house due to erosion	0.56	0.50	0.62	0.49
New families arrived	0.54	0.50	0.56	0.50
Erosion perceived as main event	0.57	0.50	0.57	0.50
N	265	265	156	156

Note: All variables except age and number of household members are dummy indicators. N for new families arrived: 126 for the full sample and 85 for the conjoint sample (due to a filter question).

Supplementary Table A.1: Summary statistics for the full sample and the sample used for the conjoint experiment

B Supplementary Discussion

B.1 Descriptive migrant attitudes including prime treatments

	Migration attitudes (PCA)	Migrants take away jobs	Migrants deserve same support	Migrants come for economic reasons	Everyone has right to settle	Migrants differ in customs and traditions
Constant	0.055	3.892	1.982	3.036	1.811	3.703
	(0.670)	(0.000)	(0.000)	(0.000)	(0.000)	(0.000)
	[−0.200, 0.311]	[3.723, 4.061]	[1.822, 2.142]	[2.829, 3.243]	[1.691, 1.931]	[3.524, 3.881]
Migration Framing	−0.085	−0.013	−0.146	−0.007	−0.054	−0.060
	(0.637)	(0.912)	(0.180)	(0.958)	(0.528)	(0.638)
	[−0.441, 0.270]	[−0.251, 0.224]	[−0.361, 0.068]	[−0.289, 0.274]	[−0.221, 0.114]	[−0.310, 0.191]
Num.Obs.	251	251	251	251	251	251
R2	0.001	0.000	0.007	0.000	0.002	0.001

Note: P-values from robust standard errors are shown in parentheses and 95% confidence intervals in square brackets.

Supplementary Table B.1: Constant and prime treatment effect for aggregate index and subcomponents of the migrant fear battery. Question wordings: “Migrants to [village name] take away jobs from the people living in [village name]”; “Migrants to [village name] should receive the same support from the local government as people living for a long time in [village name]”; “Migrants to [village name] come because of economic reasons”; “Every person has the right to settle in [village name] if he or she wants to.” “Migrants to [village name] have customs and traditions that are very different from the people in [village name].” Reply scale 1 (agree strongly) to 5 (disagree strongly).

	(1)
Constant	5.028
	(0.000)
	[4.802, 5.254]
Disputes with migrants	0.245
	(0.205)
	[−0.134, 0.623]
Num.Obs.	219
R2	0.007

Note: P-values from robust standard errors are shown in parentheses and 95% confidence intervals in square brackets.

Supplementary Table B.2: Constant and wording treatment effect for disputes in the village. Constant reports (scale 1-6) whether respondents perceive changes in dispute intensity within the community over the past year. With a wording treatment (to avoid potential spillover effects), we investigate whether these perceptions differ for disputes among natives (constant) versus those between natives and migrants (coefficient 'Disputes with migrants'). On average, respondents report a decline in overall community disputes, with a mean score of 5. Perceptions of migrant-related disputes do not differ significantly, indicating similarly low levels of conflict, and suggesting that intergroup tensions are not viewed as increasing, and communities holding low levels of migrant-related grievances.

	Acceptance as neighbors (1-5)
Constant	4.036
	(0.000)
	[3.768, 4.305]
Neighbors and marry s/o from family	0.019
	(0.920)
	[−0.359, 0.397]
Neighbors and children go to school together	0.231
	(0.184)
	[−0.111, 0.574]
Num.Obs.	165
R2	0.013

Note: P-values from robust standard errors are shown in parentheses and 95% confidence intervals in square brackets.

Supplementary Table B.3: Constant and wording treatment effect for acceptance of migrants. Question wording: "Please tell me whether you would like having people from outside (village name), meaning not from this area, as neighbors [*empty* //, and also marrying someone from your family //, whose children are going to school with your children]? Or would you dislike it, or would you not care?" Brackets denote survey-experimental insertions. Reply scale 1 (strongly dislike) - 5 (strongly like).

B.2 Additional conjoint experimental estimates and supporting information

	AMCE (w/o weights)	AMCE (with weights)
**Reason**		
family	0.144	0.119
	(0.000)	(0.089)
	[0.066, 0.221]	[−0.018, 0.255]
erosion	0.209	0.190
	(0.000)	(0.001)
	[0.138, 0.281]	[0.080, 0.300]
**Occupation**		
teacher	0.175	0.267
	(0.000)	(0.001)
	[0.108, 0.241]	[0.116, 0.418]
farmer	0.090	0.145
	(0.004)	(0.029)
	[0.028, 0.152]	[0.015, 0.276]
**Religion**		
strict muslim	0.422	0.392
	(0.000)	(0.000)
	[0.349, 0.495]	[0.284, 0.500]
lenient muslim	0.295	0.294
	(0.000)	(0.000)
	[0.221, 0.369]	[0.144, 0.445]
**Origin**		
distant	−0.056	−0.099
	(0.066)	(0.103)
	[−0.116, 0.004]	[−0.217, 0.020]
N	936	678

Note: P-values from respondent clustered standard errors are shown in parentheses and 95% confidence intervals in square brackets.

Supplementary Table B.4: Main Average Marginal Component Effects (compared to baseline levels).

	Erosion	Family	Economic
**Occupation**			
teacher	0.121	0.130	0.260
	(0.036)	(0.044)	(0.000)
	[0.008, 0.235]	[0.003, 0.257]	[0.146, 0.374]
farmer	0.062	0.061	0.155
	(0.303)	(0.349)	(0.007)
	[−0.056, 0.181]	[−0.067, 0.188]	[0.043, 0.267]
**Religion**			
strict muslim	0.433	0.442	0.403
	(0.000)	(0.000)	(0.000)
	[0.296, 0.570]	[0.313, 0.570]	[0.294, 0.512]
lenient muslim	0.211	0.366	0.334
	(0.001)	(0.000)	(0.000)
	[0.082, 0.341]	[0.243, 0.489]	[0.224, 0.445]
**Origin**			
distant	−0.009	0.034	−0.176
	(0.857)	(0.537)	(0.000)
	[−0.113, 0.094]	[−0.075, 0.143]	[−0.273, −0.078]
N	936	936	936

Note: P-values from respondent clustered standard errors are shown in parentheses and 95% confidence intervals in square brackets.

Supplementary Table B.5: Average Marginal Component Interaction Effects for occupation, religion, and origin attributes by reason (compared to baseline levels).

	Difference (erosion - economic)	Difference (family - economic)
**Occupation**		
teacher	−0.139	−0.130
	(0.095)	(0.143)
	[−0.302, 0.024]	[−0.305, 0.044]
farmer	−0.093	−0.094
	(0.294)	(0.307)
	[−0.265, 0.080]	[−0.274, 0.086]
**Religion**		
strict muslim	0.030	0.039
	(0.739)	(0.666)
	[−0.146, 0.205]	[−0.138, 0.216]
lenient muslim	−0.123	0.031
	(0.143)	(0.706)
	[−0.287, 0.042]	[−0.131, 0.194]
**Origin**		
distant	0.166	0.210
	(0.025)	(0.005)
	[0.021, 0.312]	[0.064, 0.356]
N	936	936

Note: P-values from respondent clustered standard errors are shown in parentheses and 95% confidence intervals in square brackets.

Supplementary Table B.6: Differences in Average Marginal Component Interaction Effects (Compared to Baseline Levels).

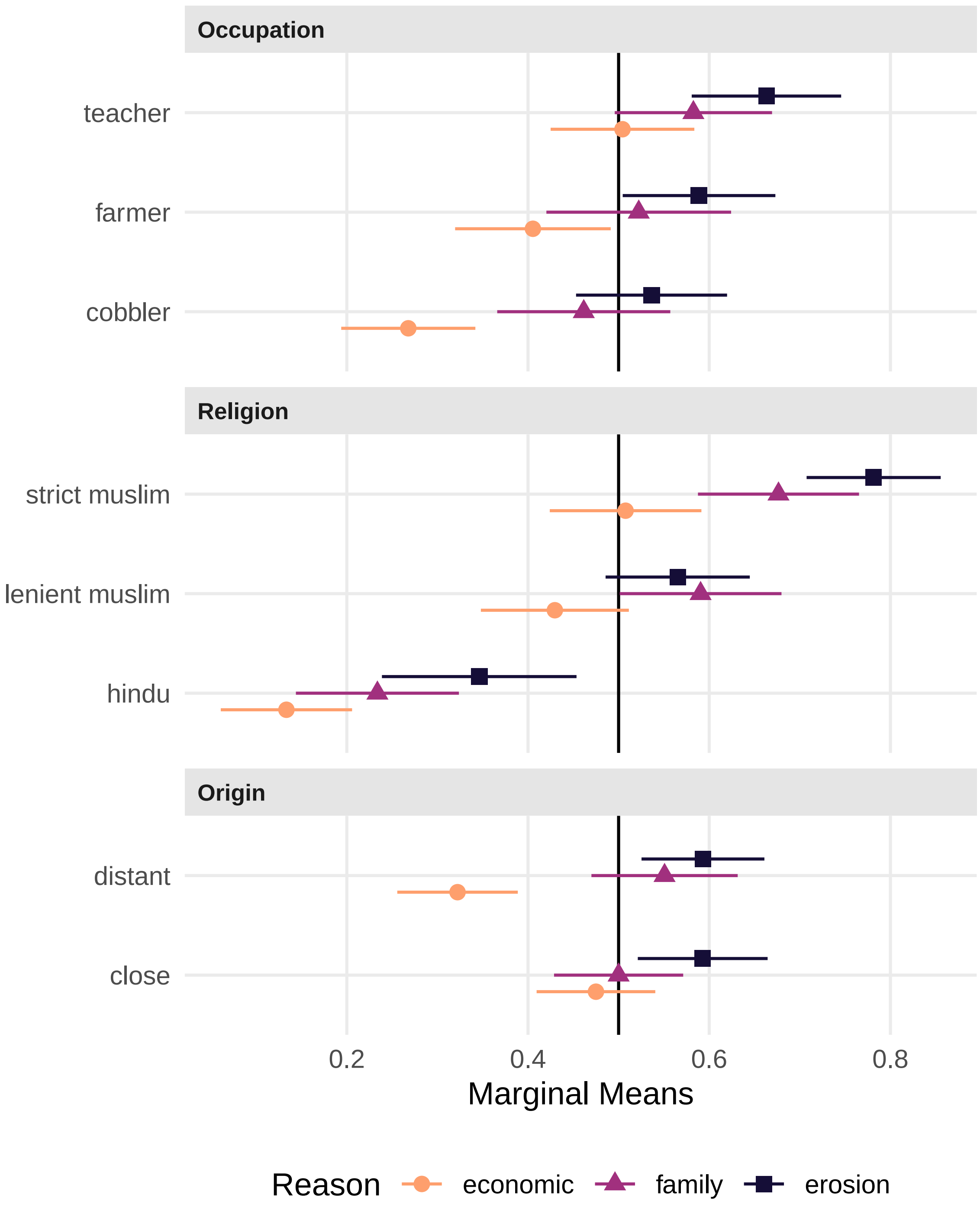

Supplementary Figure B.1: Marginal means, interacting the attribute levels of reason for migration with the remaining attributes. Orange circles denote marginal means from interactions with *economic*, purple triangles from interactions with *family* and dark purple squares from interactions with *erosion*.Error bars indicate 95% confidence intervals from respondent-clustered standard errors ($N = 936$).

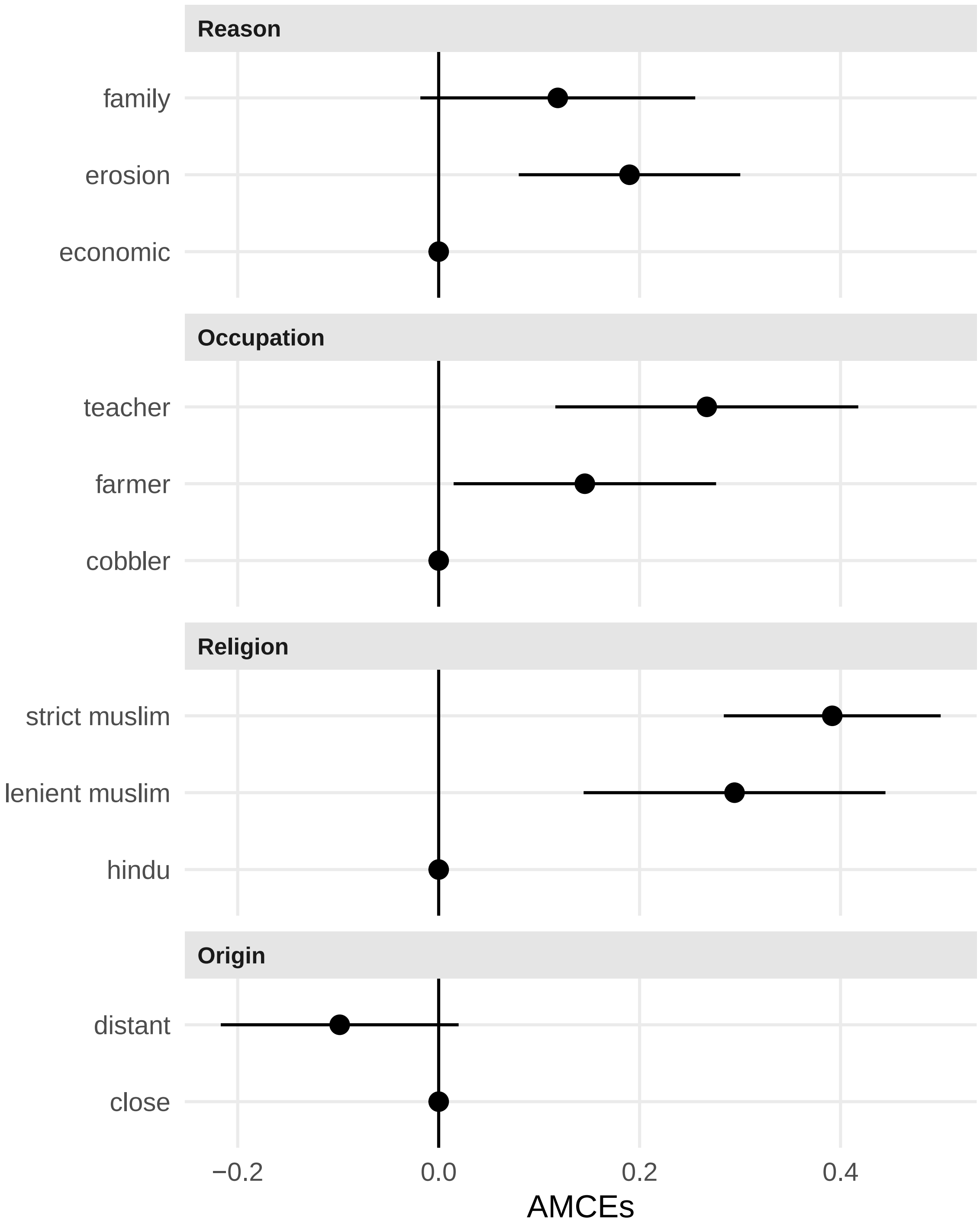

Supplementary Figure B.2: Average Marginal Component Effects for the conjoint experiment. Error bars indicate 95% confidence intervals from respondent-clustered standard errors (N = 678). Each observation is weighted for population-representativity with weights obtained from entropy balancing on the first, second, and third moments of demographics (age, marital status, gender, education, religion, household composition), income, and housing conditions (e.g., building materials, access to sanitation, water, and electricity). Numerical results are presented in Supplementary Table B.4, model 2.

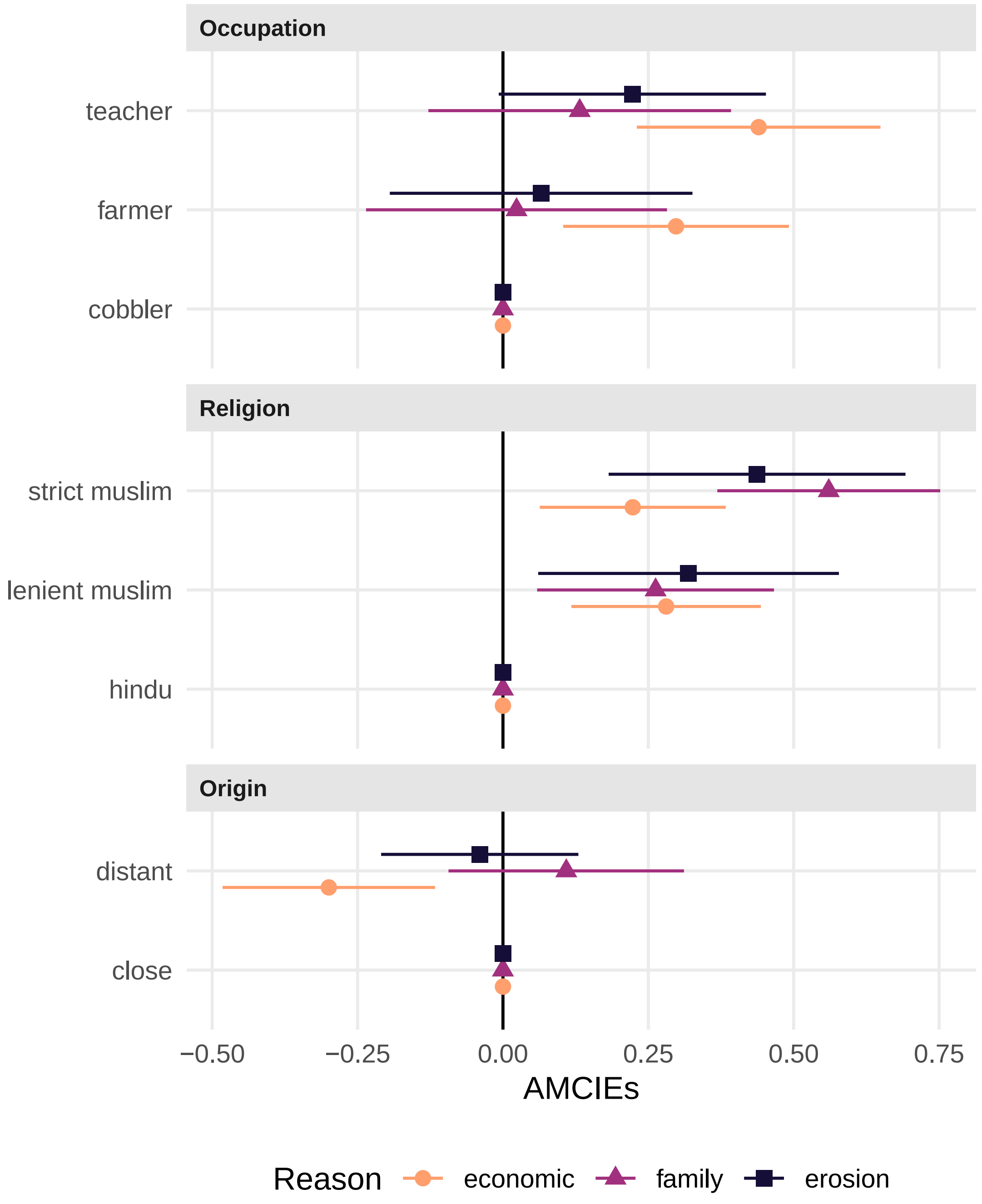

Supplementary Figure B.3: Average Marginal Component Interaction Effects (AMCIEs), interacting the attribute levels of reason for migration with the remaining attributes. Orange circles denote AMCIEs from interactions with *economic*, purple triangles from interactions with *family* and dark purple squares from interactions with *erosion*. Error bars indicate 95% confidence intervals from respondent-clustered standard errors ($N = 678$). Each observation is weighted for population-representativity with weights obtained from entropy balancing on the first, second, and third moments of demographic, economic, and housing covariates (see Supplementary Table B.9).

	Erosion Perceived as Main Event		New Families Arrived		Previously Migrated		Lost House Due to Erosion	
	Yes	No	Yes	No	Yes	No	Yes	No
**Reason**								
family	0.506	0.558	0.523	0.516	0.544	0.506	0.513	0.540
	(0.000)	(0.000)	(0.000)	(0.000)	(0.000)	(0.000)	(0.000)	(0.000)
	[0.436, 0.577]	[0.480, 0.637]	[0.422, 0.625]	[0.417, 0.615]	[0.457, 0.630]	[0.441, 0.572]	[0.445, 0.581]	[0.458, 0.621]
erosion	0.593	0.581	0.616	0.627	0.604	0.592	0.624	0.538
	(0.000)	(0.000)	(0.000)	(0.000)	(0.000)	(0.000)	(0.000)	(0.000)
	[0.524, 0.662]	[0.518, 0.644]	[0.537, 0.696]	[0.536, 0.717]	[0.533, 0.676]	[0.527, 0.656]	[0.573, 0.675]	[0.441, 0.634]
economic	0.421	0.352	0.369	0.367	0.381	0.406	0.376	0.426
	(0.000)	(0.000)	(0.000)	(0.000)	(0.000)	(0.000)	(0.000)	(0.000)
	[0.367, 0.475]	[0.284, 0.421]	[0.299, 0.438]	[0.280, 0.454]	[0.322, 0.440]	[0.344, 0.467]	[0.326, 0.427]	[0.350, 0.502]
**Occupation**								
teacher	0.596	0.551	0.547	0.589	0.580	0.578	0.577	0.580
	(0.000)	(0.000)	(0.000)	(0.000)	(0.000)	(0.000)	(0.000)	(0.000)
	[0.541, 0.650]	[0.475, 0.627]	[0.462, 0.633]	[0.488, 0.690]	[0.504, 0.657]	[0.524, 0.632]	[0.520, 0.633]	[0.508, 0.651]
farmer	0.503	0.515	0.476	0.513	0.504	0.503	0.508	0.495
	(0.000)	(0.000)	(0.000)	(0.000)	(0.000)	(0.000)	(0.000)	(0.000)
	[0.451, 0.555]	[0.453, 0.578]	[0.385, 0.568]	[0.425, 0.601]	[0.438, 0.569]	[0.452, 0.554]	[0.462, 0.554]	[0.418, 0.572]
cobbler	0.402	0.430	0.477	0.385	0.421	0.414	0.410	0.431
	(0.000)	(0.000)	(0.000)	(0.000)	(0.000)	(0.000)	(0.000)	(0.000)
	[0.355, 0.450]	[0.370, 0.489]	[0.410, 0.544]	[0.296, 0.473]	[0.367, 0.476]	[0.363, 0.465]	[0.365, 0.455]	[0.370, 0.492]
**Religion**								
strict muslim	0.645	0.659	0.638	0.675	0.623	0.674	0.652	0.642
	(0.000)	(0.000)	(0.000)	(0.000)	(0.000)	(0.000)	(0.000)	(0.000)
	[0.595, 0.696]	[0.586, 0.733]	[0.554, 0.723]	[0.575, 0.776]	[0.557, 0.688]	[0.621, 0.727]	[0.597, 0.706]	[0.578, 0.707]
lenient muslim	0.535	0.497	0.547	0.494	0.544	0.505	0.519	0.529
	(0.000)	(0.000)	(0.000)	(0.000)	(0.000)	(0.000)	(0.000)	(0.000)
	[0.479, 0.590]	[0.434, 0.559]	[0.476, 0.618]	[0.414, 0.574]	[0.478, 0.610]	[0.451, 0.559]	[0.463, 0.574]	[0.468, 0.590]
hindu	0.217	0.278	0.197	0.250	0.240	0.232	0.221	0.269
	(0.000)	(0.000)	(0.000)	(0.000)	(0.000)	(0.000)	(0.000)	(0.000)
	[0.155, 0.279]	[0.198, 0.358]	[0.113, 0.281]	[0.153, 0.347]	[0.164, 0.316]	[0.170, 0.294]	[0.156, 0.285]	[0.191, 0.347]
**Origin**								
distant	0.490	0.454	0.476	0.427	0.457	0.486	0.453	0.509
	(0.000)	(0.000)	(0.000)	(0.000)	(0.000)	(0.000)	(0.000)	(0.000)
	[0.447, 0.534]	[0.402, 0.505]	[0.421, 0.530]	[0.351, 0.503]	[0.405, 0.508]	[0.443, 0.529]	[0.411, 0.496]	[0.460, 0.557]
close	0.509	0.549	0.524	0.575	0.545	0.513	0.547	0.492
	(0.000)	(0.000)	(0.000)	(0.000)	(0.000)	(0.000)	(0.000)	(0.000)
	[0.469, 0.549]	[0.495, 0.603]	[0.471, 0.577]	[0.500, 0.651]	[0.492, 0.598]	[0.473, 0.553]	[0.504, 0.590]	[0.445, 0.538]
N	534	378	288	216	408	510	576	354

Note: P-values from respondent clustered standard errors are shown in parentheses and 95% confidence intervals in square brackets.

Supplementary Table B.7: Marginal means for subgroups of respondents as reported in Figures 3 and 4.

	Erosion Perceived as Main Event (Yes - No)	New Families Arrived (Yes - No)	Previously Migrated (Yes - No)	Lost House Due to Erosion (Yes - No)
**Reason**				
family	−0.052	0.007	0.038	−0.027
	(0.334)	(0.922)	(0.494)	(0.620)
	[−0.157, 0.053]	[−0.135, 0.149]	[−0.071, 0.146]	[−0.133, 0.079]
erosion	0.012	−0.011	0.013	0.086
	(0.802)	(0.864)	(0.795)	(0.122)
	[−0.082, 0.105]	[−0.131, 0.110]	[−0.084, 0.109]	[−0.023, 0.195]
economic	0.069	0.002	−0.024	−0.050
	(0.124)	(0.974)	(0.575)	(0.282)
	[−0.019, 0.156]	[−0.110, 0.113]	[−0.110, 0.061]	[−0.141, 0.041]
**Occupation**				
teacher	0.044	−0.042	0.002	−0.003
	(0.353)	(0.536)	(0.960)	(0.943)
	[−0.049, 0.138]	[−0.174, 0.090]	[−0.091, 0.096]	[−0.095, 0.088]
farmer	−0.012	−0.037	0.001	0.012
	(0.766)	(0.572)	(0.990)	(0.787)
	[−0.094, 0.069]	[−0.164, 0.090]	[−0.082, 0.084]	[−0.077, 0.102]
cobbler	−0.028	0.092	0.007	−0.021
	(0.478)	(0.102)	(0.849)	(0.590)
	[−0.104, 0.049]	[−0.018, 0.203]	[−0.067, 0.082]	[−0.097, 0.055]
**Religion**				
strict muslim	−0.014	−0.037	−0.051	0.010
	(0.756)	(0.578)	(0.235)	(0.825)
	[−0.103, 0.075]	[−0.169, 0.094]	[−0.136, 0.033]	[−0.075, 0.094]
lenien muslim	0.038	0.053	0.039	−0.010
	(0.370)	(0.333)	(0.374)	(0.804)
	[−0.045, 0.122]	[−0.054, 0.160]	[−0.046, 0.124]	[−0.093, 0.072]
hindu	−0.061	−0.053	0.008	−0.048
	(0.237)	(0.420)	(0.874)	(0.350)
	[−0.163, 0.040]	[−0.182, 0.076]	[−0.091, 0.107]	[−0.149, 0.053]
**Origin**				
distant	0.037	0.048	−0.029	−0.055
	(0.288)	(0.310)	(0.393)	(0.094)
	[−0.031, 0.104]	[−0.045, 0.141]	[−0.096, 0.038]	[−0.120, 0.009]
close	−0.040	−0.051	0.032	0.055
	(0.244)	(0.276)	(0.349)	(0.087)
	[−0.107, 0.027]	[−0.144, 0.041]	[−0.035, 0.098]	[−0.008, 0.119]
N	912	504	918	930

Note: P-values from respondent clustered standard errors are shown in parentheses and 95% confidence intervals in square brackets.

Supplementary Table B.8: Differences in marginal means between subgroups of respondents. Same subgroup specifications as in Figures 3 and 4.

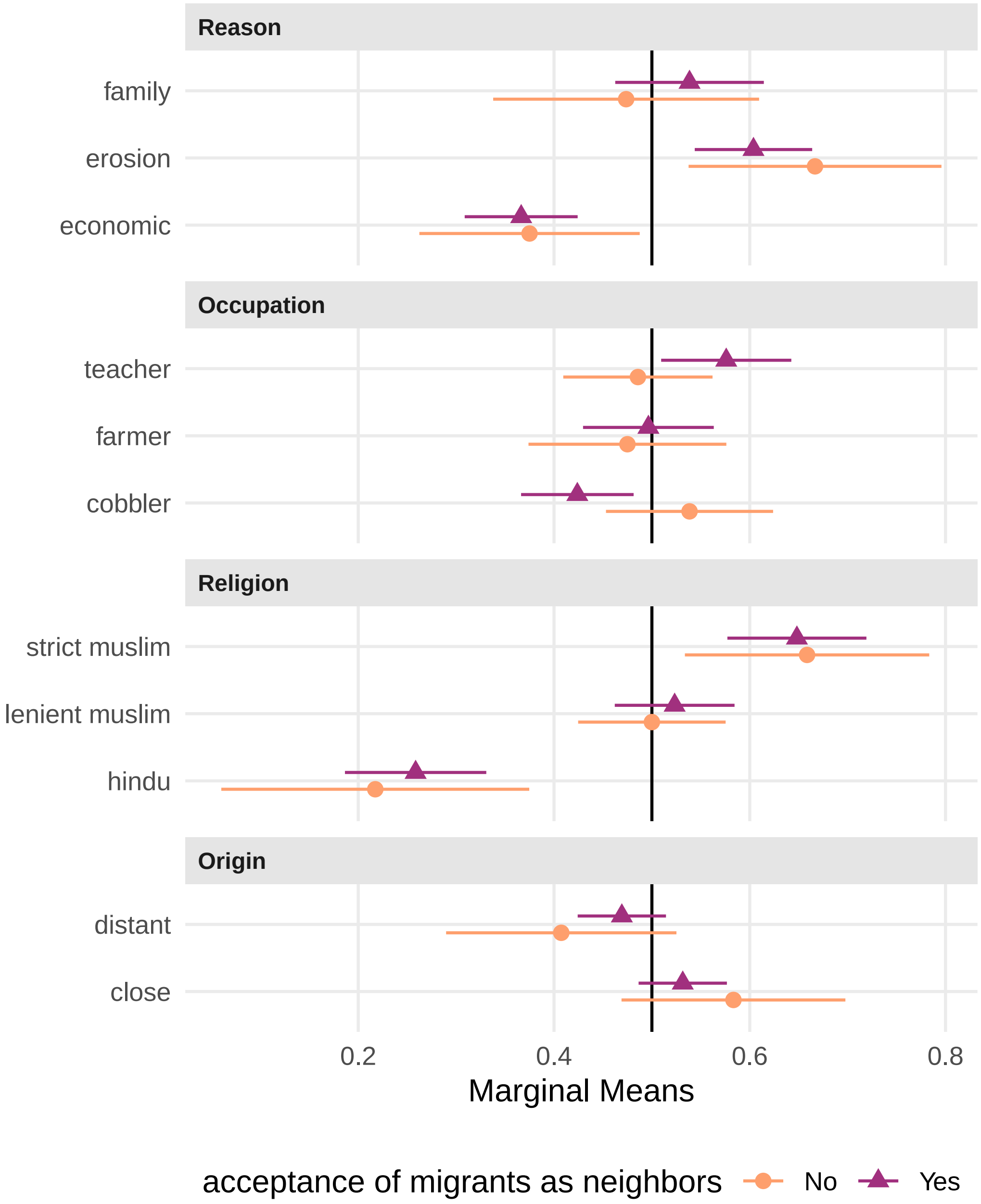

Supplementary Figure B.4: Marginal means for subgroups of respondents that said that they would accept migrants as neighbors ($N = 450$; purple triangles) vs. not ($N = 114$; orange circles). Error bars indicate 95% confidence intervals from respondent-clustered standard errors. Acceptance of migrants is measured by binarizing replies to the question "Please tell me whether you would like having people from outside (village name), meaning not from this area, as neighbors (...)" (see Supplementary Table B.3 for additional details). Respondents who said they would strongly or somewhat like migrants as neighbors were counted as accepting migrants, while those who said they would not care, somewhat dislike, or strongly dislike migrants as neighbors were categorized as not accepting migrants.

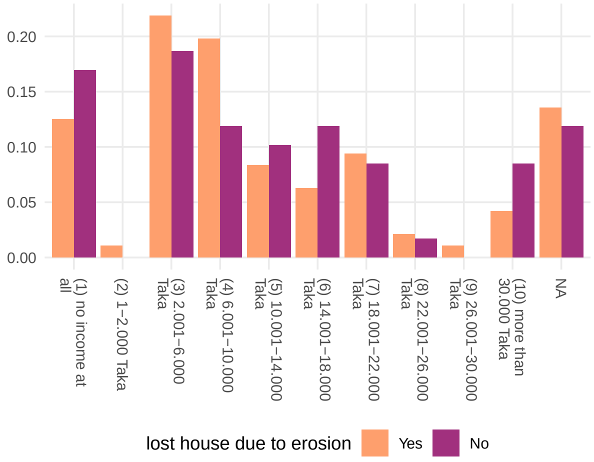

(a)

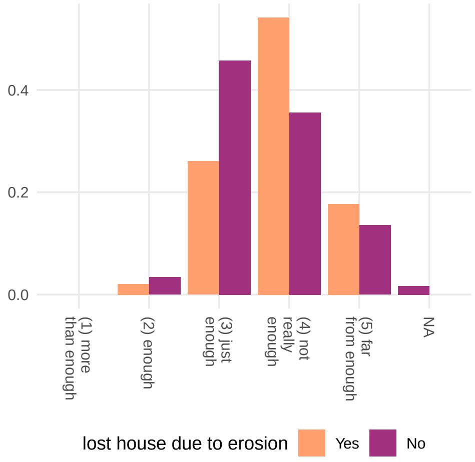

(b)

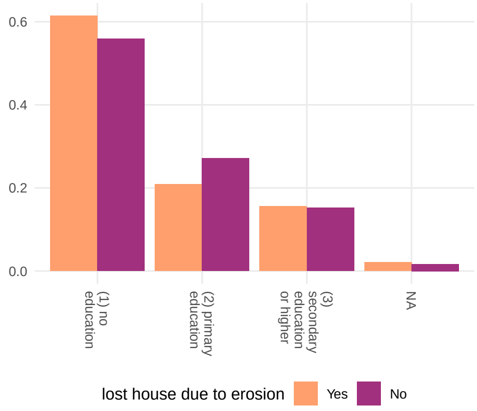

(c)

Supplementary Figure B.5: Relative frequencies of indicators of economic well-being and social class by house loss (Yes: orange shading; No: purple shading) due to erosion for the conjoint sample used in Figure 4, Panel (b) ($N_{respondents} = 155$). (a) Household income is measured on a 10-point scale. (b) Self-reported ability to sustain the household on current income is measured on a 5-point scale. (c) Education is coarsened to three levels. NAs are reported for the sake of transparency. We found no significant correlation between house loss due to erosion and household income ($r =$ ˘0.047, $p = 0.585$), a statistically significant but weak correlation between house loss and respondents' self-reported ability to sustain their household on current income ($r = 0.177$, $p = 0.028$), and no significant correlation between education and house loss ($r = -0.035$, $p = 0.667$). Correlations are estimated between a binary variable measuring house loss and the numerical values of the respective variables indicated on the x-axes (NAs excluded).

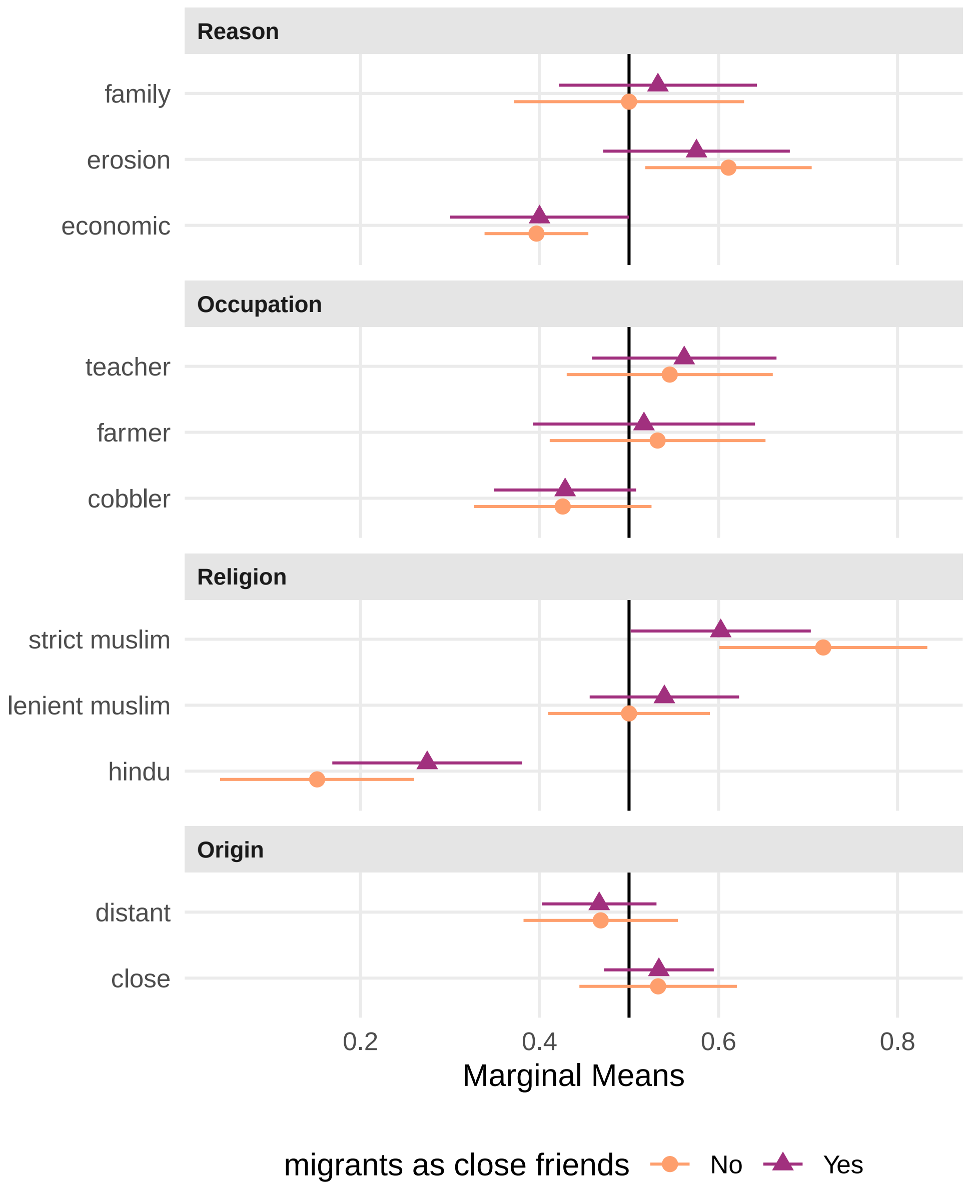

Supplementary Figure B.6: Marginal means for subgroups of respondents that have migrants as close friends ($N = 210$; purple triangles) vs. not as close friends ($N = 156$; orange circles). Error bars indicate 95% confidence intervals from respondent-clustered standard errors. The exact survey question read: "About how many of your close friends in [village] came as migrants from other places to [village] in the last 5 years?" Respondents who answered that none of their friends came as migrants were counted as having no migrants as close friends, while respondents who answered that a few, about half, many, or all of their friends have come as migrants were counted as having migrants as close friends. As this question was part of an additional priming experiment (see Methods subsection 'Additional priming experiment'), it was posed only to a random subset of the initial study sample.

Xname	mean_Tr	mean_Co_Pre	mean_Co_Post	var_Tr	var_Co_Pre	var_Co_Post	skew_Tr	skew_Co_Pre	skew_Co_Post	sdiff_Pre	sdiff_Post
age (cont.)	51.357	43.709	51.363	192.830	211.250	228.638	0.439	0.399	-0.172	0.551	-0.000
married (binary, 1=married)	0.915	0.889	0.915	0.078	0.099	0.079	-2.965	-2.475	-2.965	0.092	-0.000
sex (binary, 1=male)	0.866	0.487	0.866	0.116	0.251	0.117	-2.143	0.053	-2.143	1.110	-0.000
education = signature only (categorical, ref: none)	0.167	0.222	0.166	0.139	0.174	0.139	1.790	1.336	1.791	-0.149	0.000
education = primary (categorical, ref: none)	0.125	0.085	0.125	0.109	0.078	0.110	2.272	2.984	2.273	0.121	0.000
education = secondary (categorical, ref: none)	0.041	0.026	0.041	0.039	0.026	0.039	4.632	5.901	4.633	0.073	0.000
education = secondary passed (categorical, ref: none)	0.038	0.037	0.038	0.037	0.036	0.037	4.812	4.903	4.813	0.007	0.000
education = higher (categorical, ref: none)	0.030	0.026	0.030	0.029	0.026	0.030	5.483	5.901	5.484	0.022	0.000
religion (binary, 1=hindu)	0.017	0.011	0.017	0.017	0.011	0.017	7.491	9.566	7.493	0.049	0.000
household members below 15 (cont.)	1.357	1.407	1.357	1.244	1.275	1.524	0.768	0.531	0.726	-0.045	0.000
household members 15-65 (cont.)	3.293	3.217	3.292	2.879	2.809	2.946	1.244	1.048	0.808	0.045	0.001
household members above 65 (cont.)	0.392	0.275	0.392	0.388	0.243	0.299	1.388	1.529	0.980	0.187	-0.000
household income last month (1-2000tk) (categorical, ref: none)	0.032	0.005	0.032	0.031	0.005	0.031	5.313	13.638	5.314	0.152	0.000
household income last month (2-6000tk) (categorical, ref: none)	0.201	0.196	0.202	0.161	0.158	0.162	1.490	1.533	1.488	0.014	-0.001
household income last month (6-10,000tk) (categorical, ref: none)	0.233	0.228	0.233	0.179	0.177	0.180	1.261	1.300	1.262	0.014	0.000
household income last month (10-14,000tk) (categorical, ref: none)	0.106	0.138	0.106	0.095	0.119	0.095	2.560	2.104	2.560	-0.103	-0.000
household income last month (14-18,000tk) (categorical, ref: none)	0.063	0.085	0.063	0.059	0.078	0.060	3.589	2.984	3.590	-0.088	0.000
household income last month (18-22,000tk) (categorical, ref: none)	0.070	0.090	0.070	0.065	0.082	0.066	3.360	2.866	3.361	-0.077	0.000
household income last month (22-26,000tk) (categorical, ref: none)	0.029	0.032	0.029	0.029	0.031	0.029	5.573	5.342	5.575	-0.014	0.000
household income last month (26-30,000tk) (categorical, ref: none)	0.038	0.016	0.038	0.037	0.016	0.037	4.812	7.747	4.813	0.117	0.000
household income last month (30,000tk plus) (categorical, ref: none)	0.084	0.074	0.084	0.077	0.069	0.077	3.006	3.253	3.007	0.035	0.000
household has latrine (binary, 1=yes)	0.968	0.952	0.968	0.031	0.046	0.032	-5.313	-4.249	-5.274	0.088	0.002
house material brick (binary, 1=yes)	0.154	0.143	0.154	0.130	0.123	0.131	1.917	2.041	1.917	0.031	0.000
house material bamboo (binary, 1=yes)	0.402	0.280	0.402	0.241	0.203	0.242	0.398	0.978	0.398	0.249	0.000
house material wood (binary, 1=yes)	0.660	0.497	0.660	0.225	0.251	0.226	-0.675	0.011	-0.674	0.343	0.000
house material mud (binary, 1=yes)	0.427	0.339	0.426	0.245	0.225	0.246	0.297	0.682	0.298	0.178	0.000
house material concrete (binary, 1=yes)	0.233	0.249	0.233	0.179	0.188	0.180	1.261	1.163	1.262	-0.036	0.000
house material iron (binary, 1=yes)	0.977	0.926	0.977	0.023	0.069	0.023	-6.342	-3.253	-6.301	0.338	0.002
house material jute (binary, 1=yes)	0.055	0.048	0.055	0.052	0.046	0.053	3.895	4.249	3.884	0.033	-0.001
roof material concrete (binary, 1=yes)	0.015	0.026	0.015	0.015	0.026	0.015	7.942	5.901	7.943	-0.093	0.000
roof material steel (binary, 1=yes)	0.010	0.005	0.010	0.010	0.005	0.010	9.955	13.638	9.957	0.046	0.000
roof material tin (binary, 1=yes)	0.994	0.979	0.994	0.006	0.021	0.006	-12.547	-6.654	-12.550	0.190	-0.000
roof material wood (binary, 1=yes)	0.402	0.280	0.402	0.241	0.203	0.242	0.398	0.978	0.398	0.249	0.000
roof material hay (binary, 1=yes)	0.005	0.016	0.005	0.005	0.016	0.005	13.571	7.747	13.574	-0.144	0.000
household connected to grid (binary, 1=yes)	0.859	0.730	0.859	0.121	0.198	0.121	-2.067	-1.037	-2.067	0.371	-0.000
house has earth foundation (binary, 1=yes)	0.814	0.820	0.814	0.152	0.148	0.152	-1.613	-1.667	-1.611	-0.016	0.001

Supplementary Table B.9: Covariate means, variances, and skewness in the general riverbankside population sample (indicator: ‘Co’ in column header) and neighbor sample (indicator: ‘Tr’ in column header) and their differences pre and post weighting. $N_{populationsample} = 1123$; $N_{neighborsample} = 189$ (sample size with non-missing observations).

Estimate	Attribute	Attribute level	p-value	FDR 0.05	FDR 0.1	FDR 0.2	FDR 0.3
AMCE	reason	family	0.000	✓	✓	✓	✓
AMCE	reason	erosion	0.000	✓	✓	✓	✓
AMCE	occupation	teacher	0.000	✓	✓	✓	✓
AMCE	occupation	farmer	0.004	✓	✓	✓	✓
AMCE	religion	strict muslim	0.000	✓	✓	✓	✓
AMCE	religion	lenient muslim	0.000	✓	✓	✓	✓
AMCE	origin	distant	0.066			✓	✓
AMCIE Difference (erosion - economic)	occupation	teacher	0.095			✓	✓
AMCIE Difference (erosion - economic)	occupation	farmer	0.294				
AMCIE Difference (erosion - economic)	religion	strict muslim	0.739				
AMCIE Difference (erosion - economic)	religion	lenient muslim	0.143				✓
AMCIE Difference (erosion - economic)	origin	distant	0.025		✓	✓	✓
MM Difference (erosion perceived as main event: Yes - No)	reason	erosion	0.802				
MM Difference (new families arrived: Yes - No)	reason	erosion	0.864				
MM Difference (previously migrated: Yes - No)	reason	erosion	0.795				
MM Difference (lost house due to erosion: Yes - No)	reason	erosion	0.122			✓	✓

Supplementary Table B.10: P-values for main hypothesis tests that are reported in the article and whether p-values hold against thresholds accounting for different false-discovery rates (FDR) according to the Benjamini-Hochberg procedure. We sort p-values in ascending order, specify the total number of tests, and compare each p-value to a corresponding threshold that depends on its rank in the ordered list, the number of tests, and the specified FDR, where the FDR is the tolerated expected proportion of rejections that are type I errors (false rejections). A p-value is considered to retain significance under a given FDR level if it, or any p-value with a smaller rank (i.e., appearing earlier in the ordered list), is lower than its corresponding threshold for that FDR level.

C Supplementary Note

C.1 Deviations between pre-registration and article

We registered the pre-analysis plan with OSF, available at osf.io/nduva. The pre-registration outlines two broader research strategies and comparisons. On the one hand, a conjoint survey experiment as presented in this manuscript (research question 1 in the pre-registration). Data collection and analysis were conducted as planned and are presented in this article (note the argument was extended and supplementary tests were conducted). On the other hand, an outline of a broader comparison of preferences in migrant-receiving and non-migrant-receiving communities (research question 2 in the pre-registration). This part was largely excluded from the analysis in this article and is left to future research, as it would have entailed an additional comparison between the survey population investigated here and that of a related survey, going too far beyond the scope of a concise article. However, for reasons of completeness and transparency, we report on the results of the entailed priming experiment in Methods subsection 'Additional priming experiment' and Supplementary Table B.1.

C.2 Deviations between actual pictorial conjoint implementation and article description thereof

We note one deviation of the article from the actual survey: As described in Methods, the conjoint was presented visually to respondents. We used icons as stylized, simplified depictions of conjoint attribute levels. The pictorial icons presented in this article (Figure 5, Supplementary Figure A.1) are, however, partly replacements of the original icons with conceptually identical, but visually distinct icons. This is, as part of the icons we used we are not allowed to distribute under CC-BY licensing. While these icons are not identical to those actually presented to respondents, they are closely aligned – they explicitly represent the same underlying attributes at the same level of abstraction. The original icon representation is available from the authors on request. The full credit list for the icons, all obtained under CC-By 3.0 from The Noun Project (`https://thenounproject.com`),

for Figure 5 (top left to bottom right)/Supplementary Figure A.1 is:

- Icon with attribute name ‘Riverbank erosion’ in Fig. 5: Icon ‘Coastal erosion’, https://thenounproject.com/icon/coastal-erosion-3189069/, created by WiStudio
- Icon with attribute name ‘Family reunification’ in Fig. 5: Icon ‘Bonds’, https://thenounproject.com/icon/bonds-8227940/, created by Zpoliariumz Zydanez
- Icon with attribute name ‘Economic need’ in Fig. 5: Icon ‘Taka’, https://thenounproject.com/icon/taka-6831437/, created by BEARicons
- Icon with attribute name ‘Teacher’ in Fig. 5: Icon ‘Classroom’, https://thenounproject.com/icon/classroom-7447064/, created by Aidan Stonehouse
- Icon with attribute name ‘Farmer’ in Fig. 5: Icon ‘Planting plant’, https://thenounproject.com/icon/planting-plant-859751/, created by Gan Khoon Lay
- Icon with attribute name ‘Cobbler’ in Fig. 5: Icon ‘Cobble’, https://thenounproject.com/icon/cobble-4859933/, created by Gan Khoon Lay
- Icon with attribute name ‘Strict Muslim’ in Fig. 5: Icon ‘Veil’, https://thenounproject.com/icon/veil-2780224/, created by Izwar Muis
- Icon with attribute name ‘Lenient Muslim’ in Fig. 5: Icon ‘Hijab’, https://thenounproject.com/icon/hijab-5035617/, created by Lufti Gani Al Achmad
- Icon with attribute name ‘Hindu’ in Fig. 5: Icon ‘Indian’, https://thenounproject.com/icon/indian-3915787/, created by Phạm Thanh Lộc
- Icon with attribute name ‘Distant’ in Fig. 5: Icon ‘Bus’, https://thenounproject.com/icon/bus-1334942/, created by Adrien Coquet
- Icon with attribute name ‘Close’ in Fig. 5: Icon ‘Ricksaw’, https://thenounproject.com/icon/rickshaw-992/, created by Stephen James Kennedy
