# Supplementary material for: Perceived deservingness shapes attitudes toward environmental migrants in rural Bangladesh
Source: Commun Earth Environ. 2026 Feb 26;7(1):247. doi: 10.1038/s43247-026-03320-6 (PMC12995714; doi:10.1038/s43247-026-03320-6)
Supplement: Supplementary file 3 — Reporting Summary [file 43247_2026_3320_MOESM3_ESM.pdf]

nature portfolio

Corresponding author(s): Lukas Rudolph

Last updated by author(s): 01/10/2026

Reporting Summary

Nature Portfolio wishes to improve the reproducibility of the work that we publish. This form provides structure for consistency and transparency in reporting. For further information on Nature Portfolio policies, see our Editorial Policies and the Editorial Policy Checklist.

Statistics

For all statistical analyses, confirm that the following items are present in the figure legend, table legend, main text, or Methods section.

n/a	Confirmed	
☐	☒	The exact sample size (*n*) for each experimental group/condition, given as a discrete number and unit of measurement
☐	☒	A statement on whether measurements were taken from distinct samples or whether the same sample was measured repeatedly
☐	☒	The statistical test(s) used AND whether they are one- or two-sided<br>*Only common tests should be described solely by name; describe more complex techniques in the Methods section.*
☐	☒	A description of all covariates tested
☐	☒	A description of any assumptions or corrections, such as tests of normality and adjustment for multiple comparisons
☐	☒	A full description of the statistical parameters including central tendency (e.g. means) or other basic estimates (e.g. regression coefficient) AND variation (e.g. standard deviation) or associated estimates of uncertainty (e.g. confidence intervals)
☐	☒	For null hypothesis testing, the test statistic (e.g. *F*, *t*, *r*) with confidence intervals, effect sizes, degrees of freedom and *P* value noted<br>*Give P values as exact values whenever suitable.*
☒	☐	For Bayesian analysis, information on the choice of priors and Markov chain Monte Carlo settings
☒	☐	For hierarchical and complex designs, identification of the appropriate level for tests and full reporting of outcomes
☐	☒	Estimates of effect sizes (e.g. Cohen's *d*, Pearson's *r*), indicating how they were calculated

Our web collection on statistics for biologists contains articles on many of the points above.

Software and code

Policy information about availability of computer code

Data collection	Qualtrics survey software in combination with the Qualtrics survey offline app was used for survey data collection.
Data analysis	R and Stata were used for data analysis.

For manuscripts utilizing custom algorithms or software that are central to the research but not yet described in published literature, software must be made available to editors and reviewers. We strongly encourage code deposition in a community repository (e.g. GitHub). See the Nature Portfolio guidelines for submitting code & software for further information.

Data

Policy information about availability of data

All manuscripts must include a data availability statement. This statement should provide the following information, where applicable:

- Accession codes, unique identifiers, or web links for publicly available datasets
- A description of any restrictions on data availability
- For clinical datasets or third party data, please ensure that the statement adheres to our policy

Data and replication code are uploaded to a public repository (Harvard Dataverse), accessible via https://doi.org/10.7910/DVN/MDC2EG.

Research involving human participants, their data, or biological material

Policy information about studies with human participants or human data. See also policy information about sex, gender (identity/presentation), and sexual orientation and race, ethnicity and racism.

Reporting on sex and gender	Replies of survey respondents were not analyzed by gender, as this was not of core theoretical interest to this study.
Reporting on race, ethnicity, or other socially relevant groupings	Replies of survey respondents were not analyzed by race or ethnicity, as this was not of core theoretical interest to this study.
Population characteristics	Population characteristics are reported in SI Table A.1
Recruitment	See Methods section "Sampling strategy". We specifically targeted respondents who were neighbors of actual in-migrants, who had moved into their immediate vicinity within the 12 months prior to the survey. As part of a larger panel study, we tracked individuals who moved out from their original villages to their new destinations. After each (migrant) respondent was interviewed in their new location, survey enumerators identified and recruited nearby non-kin neighbors to participate in the conjoint experiment. Neighbors were defined as households located in the immediate vicinity, i.e., the first row of houses surrounding the migrant respondent's house. To avoid bias due to kinship ties, we excluded relatives of the migrant respondents from the sample, under the assumption that relatives would be more inclined to welcome migrants due to their family ties. For each migrant respondent, enumerators surveyed up to three neighboring respondents, depending on their availability. Eligibility for participation was limited to household heads (defined as the primary decision-maker within the household) or senior female members of the household. To caution against potential bias from sampling, we also implement a pre-registered balancing strategy, indicating representativity for the broad general rural population of the area -- conditional on a broad set of observable respondent characteristics (see Methods section "Entropy balancing for generalizability").
Ethics oversight	ETH Zurich Ethics Commission (Decision EK 2020-N-67)

Note that full information on the approval of the study protocol must also be provided in the manuscript.

Field-specific reporting

Please select the one below that is the best fit for your research. If you are not sure, read the appropriate sections before making your selection.

☐ Life sciences ☒ Behavioural & social sciences ☐ Ecological, evolutionary & environmental sciences

For a reference copy of the document with all sections, see nature.com/documents/nr-reporting-summary-flat.pdf

Behavioural & social sciences study design

All studies must disclose on these points even when the disclosure is negative.

Study description	Quantitative, survey experimental data
Research sample	A description of the study sample is provided in Supplementary Information Table A.2. The recruitment of the research sample is described in Methods section "Sampling strategy". In total, the sample comprises 265 completed interviews with villagers in rural Bangladesh, mostly proximate to the Jamuna river's eastern bank. Of these, 156 also completed the conjoint experiment (reduced sample size due to technical issues with the Qualtrics Survey App, see note in overview to pre-registration at https://osf.io/nduva). The conjoint is repeated three times with two profiles, yielding 156 * 2 * 3 = 936 observations for the conjoint experiment in total.
Sampling strategy	See above, point "recruitment" under "Research involving human participants"
Data collection	Data was collected by a local enumerator team supervised by the authors (lead: JF), with smartphones and tablets in face-to-face interviews via the Qualtrics Offline Survey App.
Timing	January - February 2024
Data exclusions	9 respondents were reported by enumerators as not having understood the conjoint task and excluded from the respective analyses.
Non-participation	100 respondents did not participate in the conjoint experiment, as technical issues with the survey app prevented conjoint data collection for the first interviews (see Methods section and pre-registration for details).
Randomization	Allocation to survey experimental groups was randomized via Qualtrics Survey software.

Reporting for specific materials, systems and methods

We require information from authors about some types of materials, experimental systems and methods used in many studies. Here, indicate whether each material, system or method listed is relevant to your study. If you are not sure if a list item applies to your research, read the appropriate section before selecting a response.

Materials & experimental systems

n/a	Involved in the study
☒	☐ Antibodies
☒	☐ Eukaryotic cell lines
☒	☐ Palaeontology and archaeology
☒	☐ Animals and other organisms
☒	☐ Clinical data
☒	☐ Dual use research of concern
☒	☐ Plants

Methods

n/a	Involved in the study
☒	☐ ChIP-seq
☒	☐ Flow cytometry
☒	☐ MRI-based neuroimaging

Plants

Seed stocks	n/a
Novel plant genotypes	n/a
Authentication	n/a
